# Testosterone and Aggressive Behavior in Man

**DOI:** 10.5812/ijem.3661

**Published:** 2012-06-30

**Authors:** Menelaos L. Batrinos

**Affiliations:** 1 Athens University Medical School, Kifisia, Greece

**Keywords:** Testosterone, Cortisol, Serotonin, Aggressiveness

## Abstract

Atavistic residues of aggressive behavior prevailing in animal life, determined by testosterone, remain attenuated in man and suppressed through familial and social inhibitions. However, it still manifests itself in various intensities and forms from; thoughts, anger, verbal aggressiveness, competition, dominance behavior, to physical violence. Testosterone plays a significant role in the arousal of these behavioral manifestations in the brain centers involved in aggression and on the development of the muscular system that enables their realization. There is evidence that testosterone levels are higher in individuals with aggressive behavior, such as prisoners who have committed violent crimes. Several field studies have also shown that testosterone levels increase during the aggressive phases of sports games. In more sensitive laboratory paradigms, it has been observed that participant’s testosterone rises in the winners of; competitions, dominance trials or in confrontations with factitious opponents. Aggressive behavior arises in the brain through interplay between subcortical structures in the amygdala and the hypothalamus in which emotions are born and the prefrontal cognitive centers where emotions are perceived and controlled. The action of testosterone on the brain begins in the embryonic stage. Earlier in development at the DNA level, the number of CAG repeats in the androgen receptor gene seems to play a role in the expression of aggressive behavior. Neuroimaging techniques in adult males have shown that testosterone activates the amygdala enhancing its emotional activity and its resistance to prefrontal restraining control. This effect is opposed by the action of cortisol which facilitates prefrontal area cognitive control on impulsive tendencies aroused in the subcortical structures. The degree of impulsivity is regulated by serotonin inhibiting receptors, and with the intervention of this neurotransmitter the major agents of the neuroendocrine influence on the brain process of aggression forms a triad. Testosterone activates the subcortical areas of the brain to produce aggression, while cortisol and serotonin act antagonistically with testosterone to reduce its effects.

## 1. Introduction

Aggressive behavior has been variously defined and it is exhibited with a broad spectrum of manifestations from the tendency to aggressiveness to physical violence. It is a primitive and common social behavior that the media report with hidden satisfaction, using it as a means of describing exciting news, and the people of civilized countries accepts its manifestation with horror and a subconscious disturbance, because such manifestations shake the comfortable belief of the difference of human conduct from that of animals. Violent and aggressive behavior is a natural and physiological element that rules animal life, driven as it is by the instincts of survival and the preservation of species through reproduction. Attenuated residues of these instincts remain in humans, albeit suppressed by familial and social inhibitions, but it still manifests in modified and various forms in accordance with the idiosyncrasy, temperament and the psychological state of each individual (1-12).

This review is a discussion of the implications of testosterone in aggressive and violent behavior, presenting the endocrine axis and the neural circuits involved in its action and focusing on the clinical aspects of the problem and neuroimaging findings. The omission of a vast body of experimental work in animals regarding the relationship of testosterone with aggression, by no means indicates a reduction of interest and esteem in the importance of these publications by the author. His intension was to review the research in the features and effects of these fundamental research findings with the physiology and psychology of man.

## 2. Relationship of Basal Testosterone and Its Fluctuations with Aggressive Behavior

Aggressiveness is exhibited in various forms and intensities from; thoughts, bodily arousal and anger to verbal, dominant, competitive traits and serious acts of violence. The manifestation of this behavioral spectrum is associated with and served by the mobilization of the muscular system. Studies of testosterone’s relationship with aggressive and violent behavior have been performed in parallel with those on the mediators of aggressive behaviors, the muscles. There is great interest in the implications of this hormone on aggression because of the social importance of this issue, it has been made more intense and vivid not only by researchers in the field, but also by other members of society such as; politicians, criminologists, law-makers, judges, police authorities and psychologists, who await informed research to define their attitudes on the basis of these studies and more specifically on the ability to answer these two questions:

a. What is the relationship of normal levels of blood testosterone and its fluctuation with aggressiveness?

b. Does the administration of hyperphysiological doses of testosterone increase aggression and violent behavior?

A positive answer was anticipated regarding the first question from extensive experimental research in animals and research conducted in prisoners. Prison is an environment in which a majority of its inmates have shown violent behavior in the past and where dominance behavior prevails. The first study in prisoners was conducted in 1972, soon after the feasibility of testosterone estimation, by Kreutz and Rosel, who found that prisoners who had committed violent crimes during their adolescence had higher testosterone levels (13). In a single sample measurement of free testosterone in the saliva of 89 prison inmates, it was found that at the extremes of the testosterone distribution, the relationship between testosterone to aggression was more striking (14). Ten out of 11 inmates with the highest testosterone concentrations had committed violent crimes, whereas 9 out of 11 who had committed non-violent crimes had the lowest testosterone levels. Similar data were reported by others (4, 15, 16), but these results should be seen with caution because of methodological limitations (small number of subjects and samples), but mainly because of the unnatural conditions of life in prison. Studies in sexual offenders independent of their brutality gave diverging results. Blood testosterone was within the normal range or increased and testosterone associated or not associated with aggression was also reported (4, 17, 18). Clinical data from the non-prisoner population necessary to confirm the above findings in normal free men is limited. Most studies have been based on self-report questionnaires, which record actual aggression and its intensity with questionable likelihood. In a series of such studies, which gave conflicting results, the majority of these confirmed the relationship of testosterone with aggressiveness reported in prisoners (4). An investigation of testosterone, cortisol and thyroxin in a sample of 4179 veterans, which has increased credibility because of its size, has shown that basal testosterone levels were positively related to antisocial and aggressive behavior (19). It is of interest, however, that supraphysiological doses of testosterone in the order of 200 mg weekly (20), or even 600 mg weekly (21), which were administered to normal men had no effect on their aggression or anger levels. Dominance and competition are also manifestations of aggressive behavior (19, 22). Man is inclined to affirm his personality by trying to be distinguished and gain influence and power in his career, in sports and in everyday life, by competing with others. Acting in a dominant way of any kind may include violent acts, as is the case in prisoners. However, these dominant traits are usually manifested by angry faces or verbal aggression in trials to dominate or to be a winner in competitive tasks (23). Physical and mental competitive tasks have been found to be associated with higher baseline values and more often with fluctuations of testosterone. Studies with a limited number of subjects have shown a positive relationship of testosterone with aggressive phases of the game in judo contests and hockey players (4, 6, 24). In dominance contests taking place in political elections, voters in democratic elections exhibit biological responses as if they had personally participated in the political contest. A small scale clinical experiment of 57 male voters in the 2008 United States presidential elections showed that saliva testosterone measured on one morning and in three evening samples remained stable in the winner’s voters indicating a resistance to the circadian decrease in the evening, whereas it dropped in those who had voted for the looser (25). In sports it has also been reported that winners have higher testosterone levels than losers (26). Fans of sporting events also experience testosterone changes (27). More sensitive manifestations to subtle aggressive stimuli are regarded to be measures of aggressiveness obtained in the laboratory through paradigms using various combinations to provoke aggressive reactions (3). In the Taylor Aggressive Paradigm (TAP) the participant competes against a factitious opponent, to whom he delivers an intense shock if he loses the trial. With this test it was reported that baseline testosterone concentrations, despite individual differences, were positively correlated with the intensity of the aggressive reaction. The Point Subtraction Aggression Paradigm (PSAP) has been used more commonly to assess the relationship between testosterone with aggressive tendencies. In PSAP, stealing money from a factitious opponent in a trial to earn money is considered to be an aggressive act as it represents intent to cause harm to the opponent. Higher baseline testosterone that was found in individuals who rejected unfair offers, was interpreted as confirming this positive relation (13). Using PASP and measuring salivary testosterone, it was demonstrated that changes in testosterone were positively correlated with aggression. It was also found that an increase in testosterone during the PASP predicted subsequent willingness to choose competitive tasks (3, 28, 29). Studies of aggressive behavior and testosterone in the delicate years of adolescence yielded conflicting results that can be understood if one takes into consideration that at each age of puberty there is great inter-individual variability of psychological maturation and an even greater variability of testosterone secretion because of the asynchronous progress of pubertal development (30).

Aggression research in human studies has revealed an interesting property of testosterone dynamics, its rapid fluctuations provoking reactive aggression in response to stimuli. It has been shown by competition paradigms in the laboratory, that short term fluctuations of testosterone were associated with eliciting aggressive behavior. These findings indicate two important features of testosterone physiology, a) changes in testosterone levels may be more important than baseline values in relation to aggressive behavior, b) and more significantly, testosterone by its rapid increase in response to a variety of stimuli, both physical and mental, is entitled to hold a position in the group of stress hormones. Rapid fluctuations of testosterone are believed to be effected by non-genomic actions, mainly through the G protein of the membrane since the DNA reaction with an androgen receptor takes time (31).

## 3. Testosterone Action on the Brain

Aggressive behavior originates in brain centers that trigger metabolic arousal of the neuroendocrine system, this leads to the expression of aggressiveness through the mobilization of the body’s muscles. The neurons of the prefrontal area, the hypothalamus and amygdala which are concerned with aggression, express significant quantities of androgen and estradiol receptors, along with the enzymes necessary for the steroidogenesis of these hormones. The local production of testosterone in neuroendocrine neurons introduces a new factor into the interpretation of the interplay of this hormone with aggressive manifestations. Locally produced testosterone and estradiol coupling with the receptors may receive a greater variety of enhancing or diminishing influences and these could modulate their effect on aggressiveness more than the testosterone produced by the Leydig cells, which are only stimulated by luteinizing hormone (LH). The degree, however, of local testosterone’s contribution to its action on the brain is not known at present, but the promptness of its production and the variety of stimuli it can receive from neighboring neurons may render it more important than the testosterone arriving to the neurons through the circulation. Testicular and locally produced testosterone multiplys, and its action is diverse due to its transformation intracellularly into dihydrotestosterone (DHT) and estradiol by the enzymes 5a-reductase and aromatase respectively, which are also expressed in the neurons associated with aggression. The testosterone metabolite androstenediol acting on GABA receptors may be another factor for the action of testosterone.

The effect of testosterone action on the brain begins in embryonic life. Testosterone receptors are expressed in the fetus earlier than the biosynthesis of testosterone which occurs in the seventh to eighth week of pregnancy. In the fifth month, the testosterone values in male fetuses reach a peack with levels approaching those of adult men. This secretory surge lasting for a few weeks inundates the brain with testosterone, inducing anatomical and organizational changes that mark the sex differentiation of the male brain in adulthood (7).

During the last two decades, the study of testosterone’s action on the brain has been revolutionized through the use of functional magnetic resonance imaging (fMRI) and positron emission tomography (PET) which has opened new horizons in the clinical investigation of brain function. With these technologies that permit the visualization and mapping of brain areas when it has been aroused metabolically in response to stimuli, hopes are born of penetrating previously unknown domains of psychic functions and emotions, aspirations similar to those expressed for the exploration of the universe after man first stepped on the moon. Case studies of patients with traumatic brain injuries or suffering from certain neurological disorders had provided vague information on the location of brain regions that might be associated with aggression. In the modern era, investigations are conducted with a higher degree of sensitivity with the aid of neuroimaging technology that permits the scanning of brain activation. A meticulous review of 17 neuroimaging studies by Buflein and Luttrell in 2005, demonstrated that the prefrontal lobe, temporal lobe and subcortical structures in the hypothalamus and amygdala are associated with aggressive and emotional behavior in a complex play of interconnections (32). The total number of subjects in these 17 publications was 532, of which 343 were studied with matched controls and the majority of the subjects were accused murderers or murderers and patients with mental diseases. Locally produced and circulating testosterone coupled with intracellular androgen receptors, reacts with the G protein of the neuron membrane and this activates the amygdala enhancing its emotional sensitivity. The amygdala plays a pivotal role in the neuroendocrine network by controlling perception and processing emotions. Cortisol receptors are also expressed in amygdala neurons as well as in serotoninergic receptors. These two chemical agents act antagonistically to testosterone in the manipulation of subcortical emotional activity and the restraining interference of the prefrontal cortex. The gonadotropin-releasing hormone (GnRH) - LH - testosterone axis is closely linked with the corticotropin-releasing hormone (CRH) - adrenocorticotropic hormone (ACTH) - cortisol axis, and each of their end products, testosterone and cortisol respectively, influences the opposite axis (11). In a pilot study of salivary testosterone and cortisol interrelationships it was found that higher testosterone levels and lower cortisol levels are associated with higher levels of anger (33). Testosterone in the hypothalamus exerts an inhibitory action on CRH and the antidiuretic hormone induces a reduction in cortisol production. More pronounced is the inhibitory effect of cortisol on GnRH. Stressful situations, such as trauma and the like, inflict significant inhibition on testosterone secretion. High testosterone levels or an increase in basal concentrations are associated with aggressive manifestations, whereas high cortisol concentrations are linked to submissive behavior. The biological balance between testosterone and cortisol has a psychological equivalent. Motivational drives are mediated by punishment and reward and expressed by approach and avoidance tendencies, sensitivity to punishment is reduced when testosterone levels increase and this means that less fear is manifested in aggressive behavior, whereas the high cortisol levels released in stressful situations increases punishment sensitivity and avoidance, resulting in the choice of flight behavior. Therefore, when a high testosterone/cortisol ratio occurs it is more likely to result in socially aggressive behavior (34). At the neuronal level of this hormonal imbalance, testosterone activates emotional processes in the amygdala increasing the resistance of this subcortical structure to prefrontal inhibiting activity and cortisol facilitates cognitive control on impulsive tendencies aroused by the emotional subcortical structures. The degree of impulsivity present also plays a significant role in the activity of the emotional subcortical brain and this adds a third factor to the testosterone-cortisol balance, serotonin (35, 36). Clinical studies have shown that the serotoninergic system regulates impulses and aggressiveness, as it has activating and inhibitory receptors in the prefrontal and the subcortical areas. Blood serotonin (5-HT) and its metabolites found in the cerebrospinal fluid are negatively related with aggression and selective 5-HT reuptake inhibitors have been shown to exhibit aggression lowering effects (37). A recent PET study In 17 males of serotonin 5-HT1a inhibitory receptors binding potential in the prefrontal area and midbrain, demonstrated a higher number of 5-HT inhibitory receptors in the prefrontal area in subjects with the higher self reported aggressive scores (38). Facial expressions of anger, a common social aggressive behavior, have also been used in protocols for studying the relationship of testosterone with aggressiveness. An investigation in a group of 21 healthy males of the influence of blood testosterone levels on amygdala activation during an emotion recognition task, demonstrated a significant correlation between testosterone and amygdala reactivity to angry and fearful faces (39). It is of interest that the impact of testosterone on the amygdala response was observed to be within the normal range of blood testosterone concentrations. Recently a new influencing factor on aggressive behavior has emerged: the structural quality of the androgen receptor. The length of the trinucleotide CAG repeats in the promoter region of the human androgen receptor has been found to be associated with aggressive behavior. In a large scale study in India reported by Rajender et al., 241 men convicted for rape, 107 for murder and 26 for murder and rape had significantly shorter CAG repeats than the 271 male controls (40). Free testosterone was also found to be more positively related to aggressive risk taking in 301 adolescents with shorter CAG repeat length (38). Reactivity of the ventral amygdala assessed by fMRI was lowest among men with a larger number of CAG repeats (41). The clinical implications, however, of these and other studies of the genetics of human aggression is too early to be fully evaluated (42). The theory emerging from these studies is that prefrontal sections are centers which control the emotional signals coming from interconnected subcortical structures, by imposing a restraining effect to them. Disorders of these centers that diminish their function leave subcortical activity uncontrolled to express aggressive behaviors. In simplistic phrasing, the conditions for manifesting aggression are either a diminished functioning of the prefrontal cortex in relation to subcortical structures or an increased activity of these structures in relation to the prefrontal cortex. Testosterone, cortisol and serotonin are the major agents influencing this process, with testosterone activating aggression reactions and cortisol and serotonin acting antagonistically to testosterone to reduce its effect.

## 4. Conclusions

The goal of this review was to highlight the clinical data and the neuroimaging findings in man concerning the relationship between testosterone and aggressive behavior. Atavistic residues of aggressive behavior prevailing in animal life determined by testosterone remain in man, attenuated and suppressed by familial and social inhibitions, but still manifesting in various intensities and forms from thoughts, anger, verbal aggressiveness, competition, dominance to physical violence. Testosterone plays a significant role in the arousal of these behavioral manifestations in the brain centers involved in aggression and on the development of the muscular system that effects their realization. There is evidence that testosterone levels are higher in individuals with aggressive behavior, such as prisoners who have committed violent crimes. Several field studies have also shown that testosterone increases during the aggressive phases of sports games. Most of the studies, however, were conducted by self reported questionnaires, the accuracy of which is questionable. In more sensitive laboratory paradigms it was observed that a participant’s testosterone rises in the winners of competitions and dominance trials, or in confrontations with factitious opponents. This created the theory that fluctuations of testosterone may be more significant than basal values in the importance of testosterone estimation in relation to aggression. On the other hand, the rapid increase of testosterone in the above situations entitles testosterone to be characterized as a stress hormone. All the above studies have methodological limitations because of the small number of subjects and samples. More creditability comes from a large survey conducted on 4179 normal men which showed higher normal values in subjects with aggressive personality or antisocial conduct (25). It is of interest, however, that the administration of high doses of testosterone in normal men had no effect on the self reported aggression scores of the subjects.

Aggressive behavior arises in the brain through interplay between the subcortical structures in the amygdala and the hypothalamus in which emotions are born and the prefrontal cognitive centers where emotions are perceived and controlled. Locally produced testosterone is assumed to be more important in the process of aggressive arousal than testicular testosterone arriving in the circulation. The action of testosterone on the brain begins in embryonic life. During the fourth to fifth month of pregnancy a surge of fetal testosterone occurs reaching adult testosterone levels which induces anatomical and organizational changes in the male embryos brain. Even earlier at the DNA level, the number of CAG repeats in the genes of the androgen receptors appear to play a role in the expression of aggressive behavior. Men with fewer CAG repeats have more active androgen receptors and enhanced testosterone action. Asynchronous psychological maturation and the variability of testosterone secretion depends on the stage of puberty, and this explains the inconsistent and conflicting results of the studies of testosterone relationship with aggression in adolescents. In adult males neuroimaging techniques that have permitted visualization of brain functions have shown that testosterone activates the amygdala enhancing its emotional activity and its resistance to prefrontal restraining control. This effect is opposed by the action of cortisol which facilitates prefrontal area cognitive control on impulsive tendencies aroused in the subcortical structures. The degree of impulsivity is regulated by serotonin inhibiting receptors. The major agents of neuroendocrine influence on aggression in brain process form a triad: testosterone activates subcortical tendencies towards aggression and cortisol and serotonin act antagonistically to testosterone.
